# An acquired plica-induced notch in the medial femoral condyle in a patient with medial patellar plica syndrome: a case report

**DOI:** 10.1186/s12891-021-04183-y

**Published:** 2021-03-24

**Authors:** Sung-Jae Kim, Yong Gon Koh, Yong Sang Kim

**Affiliations:** Department of Orthopaedic Surgery, Center for Stem Cell & Arthritis Research, Yonsei Sarang Hospital, 10, Hyoryeong-ro, Seocho-gu, Seoul, 06698 Republic of Korea

**Keywords:** Medial patellar plica, Articular surface, Cartilage damage, Medial femoral condyle, Knee

## Abstract

**Background:**

An inflamed and thickened medial patellar plica (MPP) caused by repeated mechanical irritation from trauma or overuse leads to impingement between the anterior medial femoral condyle and the medial articular facet of the patella and produces pain or clicking, which is known as MPP syndrome. In patients with MPP syndrome, cartilage damage may occur depending on the shape of the MPP and the duration of the impingement.

**Case presentation:**

Preoperative magnetic resonance imaging in a 17-year-old male patient with MPP syndrome showed a hypertrophic MPP along with an abnormal notch in the articular surface of the medial femoral condyle. We considered that the impinged hypertrophic plica between the anterior medial femoral condyle and the medial articular facet of the patella resulted in cartilage damage on the articular surface of the medial femoral condyle. However, during arthroscopic surgery, we found that the cartilage of the notch, which was located beneath the MPP, was completely intact. We concluded that this abnormal notch had developed gradually in the MPP without cartilage damage.

**Conclusions:**

Surgeons should be mindful that acquired plica-induced notches in the articular surface of the medial femoral condyle can present in patients with MPP syndrome.

## Background

Synovial plicae in the knee joint are remnants of divisions between the compartments that are present in the knee during embryological development [1]. They are classified according to their corresponding anatomical site, and are therefore known as suprapatellar, infrapatellar, medial, and lateral patella plicae [2]. The presence of plicae at all these anatomical locations is an anatomical variant and not considered pathological [2]. Among these plicae, a medial patellar plica (MPP) has been reported in many healthy knees, and it is asymptomatic in most cases [3]. The MPP originates along the medial wall of the joint, runs obliquely downwards, and merges with the synovial membrane covering the infrapatellar fat pad [4]. Knee overuse and trauma may cause MPP to become inflamed and thickened [5]. The MPP syndrome is characterized by painful clicking of knee joint caused by the impingement of thickened MPP [6, 7]. The articular cartilage pathology associated with MPP syndrome has been reported by several authors [7–10]. The cartilage damage in patients affected by MPP syndrome usually occurs in the area of the antero-medial femoral condyle and the medial articular facet of the patella, suggesting that such damage is caused by the prolonged impingement of the plica against this aspect of the femur and the patella [8].

Here, we present a case of MPP syndrome showing a hypertrophic MPP along with an abnormal notch in the articular surface of the medial femoral condyle, which was assumed to be a damaged cartilage lesion, on preoperative magnetic resonance imaging (MRI).

## Case presentation

**Fig. 1:**
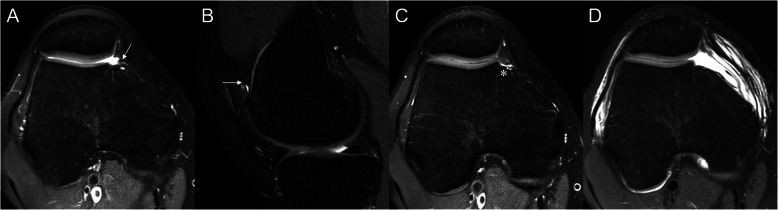
Preoperative axial (**a**) and sagittal (**b**) proton density-weighted images showing medial patellar plica (white arrows). **c** Idiopathic groove is observed in the articular surface of the medial femoral condyle (white asterisk). **d** Postoperative axial proton density-weighted image after excision of the medial patellar plica

A 17-year-old male patient was referred to our clinic for recurrent right knee pain with snapping that occurred during active extension and prevented the patient from completely extending his knee. However, when the knee was in a relaxed position, it could be extended fully by another person. The patient reported no recent traumatic events or previous surgeries. Physical examination showed a positive sign in the MPP test [11], which was conducted with the patient in the supine position and with the knee extended. Using the thumb, force was applied to press the inferomedial portion of the patellofemoral joint, inserting the medial plica between the medial femoral condyle and the patella. While maintaining this force, the knee was flexed at 90°. The MPP test was defined as positive when the patient experienced pain with the knee in extension and eliminated or markedly diminished pain with the knee in 90° of flexion. In the present case, the pain subsided when the knee was in hyperextension. A tender band was palpable approximately a fingerbreadth medial from the patella, while rolling over the medial femoral condyle was palpable with the knee in motion (the rolling over sign). Preoperative MRI was performed, which revealed a hypertrophic MPP (Fig. 1a and b) along with an abnormal notch in the articular surface of the medial femoral condyle (Fig. 1c).

**Fig. 2:**
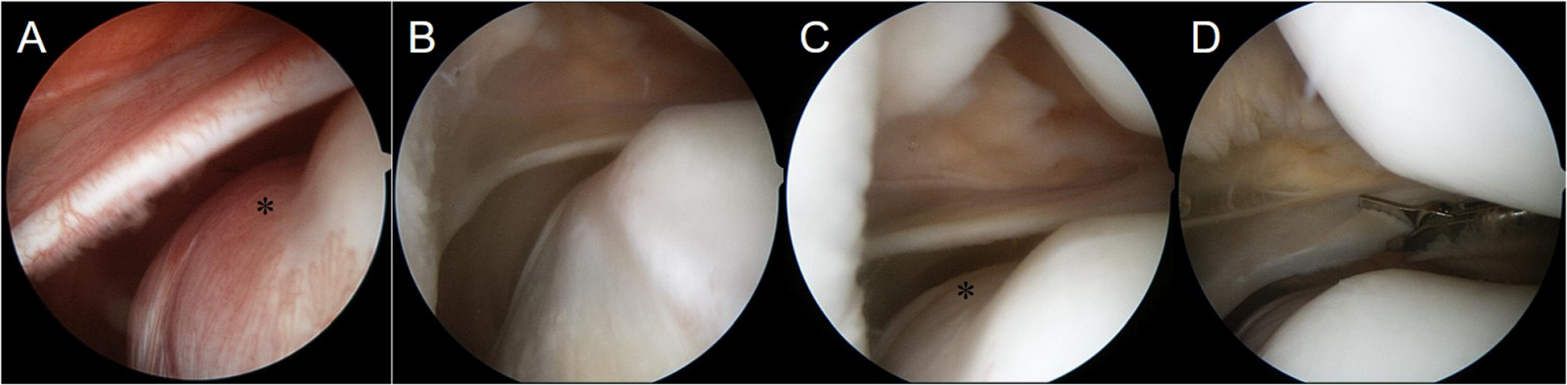
Intraoperative arthroscopic views. **a** Medial patellar plica (MPP) with a tight and hypertrophic margin is observed, along with an abnormal notch in articular surface of the medial femoral condyle beneath the MPP (black asterisk). **b** The MPP runs downwards and merges with the synovial membrane covering the infrapatellar fat pad. **c** The MPP becomes impinged in the patellofemoral joint above an abnormal notch of the medial femoral condyle (black asterisk). **d** The MPP is excised with scissor forceps

**Fig. 3:**
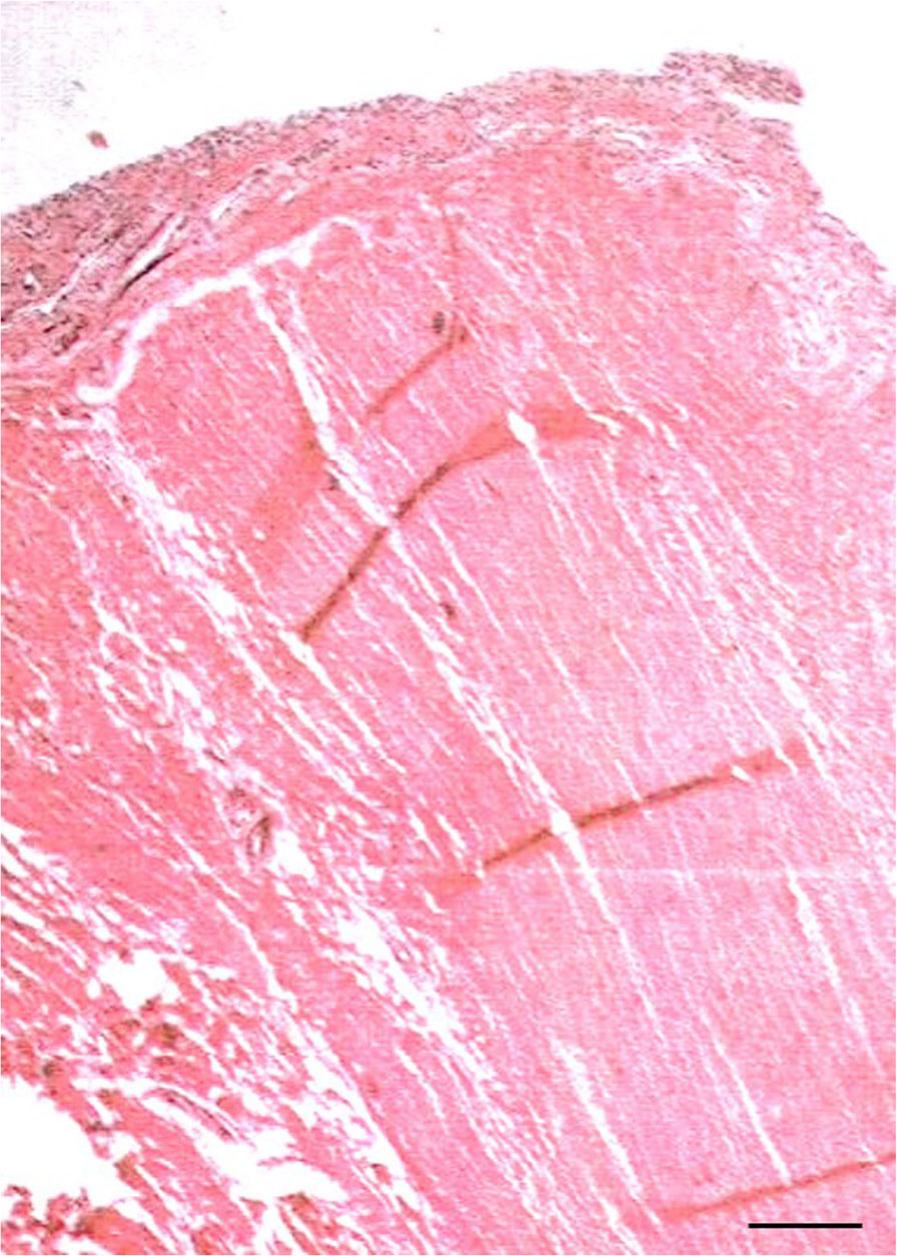
Histological findings of the resected medial patellar plica stained with hematoxylin and eosin. Scale bars = 2 mm. Fibrosis (dense fibers) are observed, and edema-like stroma can be seen in some parts. Infiltration of inflammatory cells was observed in fibrous tissues covered with synovial membrane cells

Arthroscopic surgery was performed. An MPP with a tight and hypertrophic margin was observed (Fig. 2). The position and possible impingement of the plica were observed during knee motion and checked using an arthroscopic probe. An abnormal notch in the articular surface of the medial femoral condyle was observed beneath the MPP (Fig. 2). The plica was excised at the zone of contact with the patella and medial femoral condyle (Fig. 2). The cartilage was assessed using probe palpation, which revealed no pathological lesions. Full range of motion of the knee joint and full weight-bearing were allowed immediately after surgery. One day after the surgery, postoperative MRI confirmed MPP excision (Fig. 1d), and histologic examination using a light microscope (Olympus BX53, Japan) was performed. Histological findings revealed generalized fibrosis with stroma, as well as infiltration of inflammatory cells into fibrous tissues covered with synovial membrane cells (Fig. 3).

Before surgery, the patient presented with pain (visual analogue scale [VAS] score: 5) in the medial margin of the patella and complained of palpable snapping during knee flexion and extension. The patient visited the clinic 2 weeks after the surgery and was satisfied with the improvement in his pain (VAS score: 0).

## Discussion and conclusions

MPP syndrome is difficult to diagnose, because its symptoms mimic those of many problems that cause internal derangement of the knee [5]. The diagnosis is therefore based on nonspecific symptoms such as intermittent, dull, aching pain in the area medial to the patella above the joint line and in the superomedial patellar area [12]. Rovere and Adair [13] reported that an MPP can be confirmed using physical examination if the patient shows (1) positive palpation of a tender, thickened MPP in the absence of significant effusion, and (2) no other causes of knee pain. Kim et al. [11] described the MPP test, which has shown high sensitivity and specificity for detecting MPP syndrome [14]. In the present case, the patient showed a positive MPP test result, and a hypertrophic MPP was observed on preoperative MRI (Fig. 1a and b). No other pathological lesions were found on preoperative MRI. However, an abnormal notch in the articular surface of the medial femoral condyle was observed (Fig. 1c). Before arthroscopic surgery, we assumed that this abnormal notch was a damaged cartilage lesion, because plica and cartilage damage are reportedly associated [7–9]. Christoforakis et al. [7] reported that synovial plicae of the knee are associated with an increased incidence of cartilage lesions and patients with plicae show an increased incidence of chondral lesions, particularly in the lower patella and non-weight-bearing medial femoral condyle. Kan et al. [8] reported that the shape of the medial synovial plica was associated with cartilage damage, as was the duration between symptom onset and surgery. They also concluded that surgical treatment should be considered when the medial synovial plica covers part of the anterior aspect of the medial femoral condyle, when it ruptures, or when pain persists over a long period. In such cases, surgery reduces the potential for cartilage damage. Therefore, in the present case, we considered that the hypertrophic plica was impinged between the anterior medial femoral condyle and the medial articular facet of the patella, resulting in cartilage damage on the articular surface of the medial femoral condyle. However, during arthroscopic surgery, we found that the cartilage of the notch, which was located beneath the MPP, was completely intact (Fig. 2). We concluded that this abnormal notch had developed gradually in the MPP without cartilage damage. In patients with MPP syndrome, the MPP is impinged in the patellofemoral joint, causing typical anteromedial knee pain accompanied by palpable snapping during flexion. This can cause cartilage damage of the medial femoral condyle [8, 9, 15]. Lyu [9] investigated the kinematic relationship between the medial plica and medial femoral condyle during knee motion, revealing that the pattern of medial–lateral motion can generate shear force that acts on the cartilage of the medial femoral condyle. However, in the present case, the MPP did not induce cartilage damage, but caused an abnormal notch in the medial femoral condyle. We cannot explain the exact mechanism underlying this, but we speculate that the MPP contributed to the development of an abnormal notch as this patient grew. In addition, upon physical examination, the MPP test showed a positive result, indicating that the patient experienced pain with changes in the position of the knee from flexion to extension. However, the pain subsided when the knee was in hyperextension. We considered that the pain may have subsided as the impinged MPP escaped through the notch in the medial femoral condyle.

In summary, surgeons should be mindful that acquired plica-induced notches in the articular surface of the medial femoral condyle can present in patients with MPP syndrome.

## Data Availability

The datasets used and/or analysed during the current study are available from the corresponding author on reasonable request.

## Abbreviations

MPP: Medial patellar plica
MRI: Magnetic resonance imaging
VAS: Visual analog scale

## References

[1] Ogata S, Uhthoff HK (1990). The development of synovial plicae in human knee joints: an embryologic study. Arthroscopy..

[2] Nakayama A, Sugita T, Aizawa T, Takahashi A, Honma T (2011). Incidence of medial plica in 3,889 knee joints in the Japanese population. Arthroscopy..

[3] Dandy DJ (1990). Anatomy of the medial suprapatellar plica and medial synovial shelf. Arthroscopy..

[4] Patel D (1978). Arthroscopy of the plicae--synovial folds and their significance. Am J Sports Med.

[5] Klein W (1983). The medial shelf of the knee. A follow-up study. Arch Orthop Trauma Surg.

[6] Schindler OS (2014). ‘The sneaky Plica’ revisited: morphology, pathophysiology and treatment of synovial plicae of the knee. Knee Surg Sports Traumatol Arthrosc.

[7] Christoforakis JJ, Sanchez-Ballester J, Hunt N, Thomas R, Strachan RK (2006). Synovial shelves of the knee: association with chondral lesions. Knee Surg Sports Traumatol Arthrosc.

[8] Kan H, Arai Y, Nakagawa S, Inoue H, Hara K, Minami G, et al. Characteristics of medial plica syndrome complicated with cartilage damage. Int Orthop. 2015;39(12):2489–94. 10.1007/s00264-015-2782-y. 10.1007/s00264-015-2782-y 25900367

[9] Lyu SR (2007). Relationship of medial plica and medial femoral condyle during flexion. Clin Biomech (Bristol, Avon).

[10] Arøen A, Løken S, Heir S, Alvik E, Ekeland A, Granlund OG, et al. Articular cartilage lesions in 993 consecutive knee arthroscopies. Am J Sports Med. 2004;32(1):211–5. 10.1177/0363546503259345. 10.1177/0363546503259345 14754746

[11] Kim SJ, Jeong JH, Cheon YM, Ryu SW (2004). MPP test in the diagnosis of medial patellar plica syndrome. Arthroscopy..

[12] Sznajderman T, Smorgick Y, Lindner D, Beer Y, Agar G (2009). Medial plica syndrome. Isr Med Assoc J.

[13] Rovere GD, Adair DM (1985). Medial synovial shelf plica syndrome. Treatment by intraplical steroid injection. Am J Sports Med.

[14] Kim SJ, Lee DH, Kim TE (2007). The relationship between the MPP test and arthroscopically found medial patellar plica pathology. Arthroscopy..

[15] Shetty VD, Vowler SL, Krishnamurthy S, Halliday AE (2007). Clinical diagnosis of medial plica syndrome of the knee: a prospective study. J Knee Surg.

